# Pyorrhœa Alveolaris, Etiology and Treatment

**Published:** 1888-09

**Authors:** J. D. Patterson


ARTICLE II.

### PYORRHŒA ALVEOLARIS, ETIOLOGY AND TREATMENT.

BY J. D. PATTERSON, D. D. S.

At the annual meeting of this association in 1884 I presented to the profession a study on "The Catarrhal Nature of Pyorrhœa Alveolaris," giving a history of cases in my practice confirmimg the identity of the disease with catarrh found on other mucous surfaces within the body. I am gratified at this time to present some further observations to the association which received my remarks in 1884 with such complimentary attention.

At the meeting of the American Dental Association following, at Minneapolis, in 1885, I again read an essay on the subject, giving continued experience in proving the identity of the two diseases. Since that time I have given the third article upon the subject to the "Annual of the Universal Medical Sciences," and its conclusions are embodied in Volume IV., pp. 465, 466, 467, 468, of that work.

I will not detain you by rehearsing what I have already published upon the subject, because it is well known to most of you, and I desire at this time to add instead continued study of the disease; however, I will very briefly refer to past published records. Up to the time covered by the present paper I had had under my care, since giving special attention to the disease, commencing about the year 1882, thirty-eight cases of well-marked pyorrhœa, thirty-three of which presented undoubted evidence of catarrhal conditions of the nasal or pharyngeal passages. I have maintained, and do still maintain, that catarrh; as known to physicians and surgeons, to which all mucous surfaces are exceedingly prone, exhibits closely every important pathological phase in appearance and effect to the disease we have called pyorrhœa alveolaris; and that differences found are merely the natural result to be expected on account of local anatomical forms different from other structures where is exhibited the well-known catarrhal peculiarities.

I have heretofore shown—

1st. The similar appearance of the affected mucous membrane in both diseases, and in the various stages of each.

2d. The identical character of the effusions; first being serous, containing numerous epithelial scales, and afterwards being filled with pus corpuscles and blood corpuscles.

3d. The infectious character of both diseases.

4th. The burrowing of pus, similar in both.

5th. The tendency to destruction of underlying bony tissue.

6th. The similar deposits thrown down in both diseases.

I have also claimed that catarrh is often present in the oral cavity without contagion from related mucous surfaces; also that it is resultant from contagion from the nasal passages or pharynx; that it is the result also of the interruption of the proper physical function in respiration.

I have often found difficulty in explaining this point so as to be rightly understood, and I desire to state it again. For instance, from one of the innumerable causes of nasal catarrh we have the characteristic nasal conditions, ending hyperæmia, then the resulting hypertrophy; now we find the usual diameter and capacity of the air-passages lessened—a stenosis—and for relief the sufferer becomes the well-known and much-to-be-deplored "mouth-breather." When mouth breathing is instituted and left uncombated, nothing outside of a miracle can prevent the mucous surfaces in the oral cavity from becoming catarrhal. In mouth-breathing the natural condition of the gums is rapidly changed, the fluids with which they are lubricated are dried up their outlets closed, foreign spores and unnatural thermal conditions are introduced, and catarrh of the gums—pyorrhœa, as all have been accustomed to term it—ensues in all its various and disagreeable phases.

Before proceeding farther I will give the history of cases which as yet I have not publicly reported. I have usually a small degree of credit to give to the fragmentary statement of a few or individual cases, because almost anything can be proved by cases in practice, but at this time I conclude the careful record of so many cases—which record includes every case coming under my notice that with the present knowledge of the subject would strictly be called pyorrhœa alveolaris—that I believe they must present to your minds, as they do to mine, good evidence of my claim that the disease is properly denominated and considered as an "oral catarrh"; that they are valuable, as all carefully conducted statistics must be, in deciding upon certain mooted questions all over the scientific world.

CASE I.—January, 1888, Mr. S., æt. 26. All the teeth affected, two deep pockets between molar and bicuspid on each side; patient healthy, temperament sanguine lymphatic; asserted no catarrh, but examination of nasal passages proved its presence; anterior teeth prominent, and not easily covered with the lips.

CASE 2.—February, Master., G. æt. 17. Came to have anterior teeth cleaned and filled; superior bicuspids on each side affected, pockets extending only a short distance; labio-cervical aspect of anteior teeth stained badly; sleeps with mouth open; catarrh in nose, and had received treatment for same.

CASE 3.—February, Mrs. B., æt. about 25. Front incisors very long; gums receded; difficult to keep teeth covered with lips; an evident mouth-breather; had been under treatment for catarrh; several bad pockets.

Do not expect anything and do not promise anything in this case save temporary relief. After treating, placed a retaining appliance on all teeth back to the molars, lower and upper jaws, which I apprehend will have to be worn always.

CASE 4.—Miss R., æt. about 25. Gums affected around all remaining teeth, there being ten in upper and eight in lower jaws; one deep pocket in anterior prox. surface of cuspid root, caused, so far as I could determine, by calcic inflammation.

CASE 5.—Mrs. C., æt. about 28. All the teeth affected primarily with calcic inflammation, developing into pyorrhœa; had been under treatment; four lower incisors especially loose and ready to drop out. After treatment, inserted retaining appliance for the lower incisor, extending to the bicuspids on each side, holding the teeth firmly in position.

CASE 6.—Mrs. M., æt about 27. Complete set in place; anterior teeth prominent, but generally regular; in first stages of pyorrhœa; gums irritated and red at gingival margin; no calculus, but lack of cleanliness; teeth at gum margin very sensitive; gums loose to margin of alveolus. Diagnosis: Function destroyed by want of care and by mouth-breathing; lips do not cover tips when at rest.

RECAPITULATION.—Up to conclusion of this fourth paper upon the subject,. I have now had forty-four cases, of which thirty-six exhibited catarrh in nasal passages, the remaining eight caused by mouth-breathing, calcic inflammation, or partial dentures.

Now whatever may be the inception of the disease—and I think this is generally easily ascertained—it is still not inappropriate to use the term "oral catarrh" in designating it. For instance, the inflammation may be caused entirely by calcic deposits; it may be in the first place simply salivary calculus, yet the calculus has only been the initiative step, and, if removed, the disease is, on account of the destroyed gum attachment, afforded a starting point, which now goes on as pyorrhœa, or, as I would say, an "oral catarrh"—yet it is thus properly named. Catarrhs of the nose have their origin in numerous ways—from coryza, from thermal change, from spores in inspired air, from a lack of use, as in mouth-breathing, from irritants mechanically introduced—yet their effects are classed under the one head of "catarrh." So I am of the opinion, should this oral catarrh cover all the mouth conditions where is exhibited the classical catarrhal peculiarities, whatever may have been the cause of the original changing of the proper function at the gingival margin of the gum. Here is the commencement: some irritative whereby the integrity of the dentium ligamentum is destroyed. There can be no pyorrhœa otherwise, it is impossible. My experience and observation teach me that the principal cause of the trouble is found in catarrh in the adjoining passages, communicating to the mouth by contamination in the irritative secretions, or by physical habits induced by the catarrh, or by common origin in the mouth, as in the nasal passages. The term "catarrh" means this, and this only—"I flow" or "I flow down," and is "a discharge of fluid from a mucous membrane" (Webster, Dunglison, Harris); not—observe—a discharge of fluid from the nose, the fauces, the bronchia, or the stomach, but a mucous membrane, or, as the French put it, "any mucous membrane" (Dunglison). Now, in all candor, why is pyorrhœa not a catarrh in the light of these plain, unmistakable definitions, and when too, we see all the different pathological changes in the disease exactly reproduced in catarrh? From the beginning we find in pyorrhœa "a fluid discharge from the mucous membrane," we find hyperæmia, we find hypertrophy. Then why is it not catarrh? We find catarrh in every part of the body covered by mucous membrane. Then, when it appears in the oral cavity, why must we call it something else? Why must the large territory of the oral cavity covered by mucous membrane and especially liable to irritation, why doubt that it, too, will become the seat of catarrh? The medical diagnosticans have called catarrh catarrh, whether in the nose or in the bladder, if the symptoms were identical, so as to insure definiteness and be rid of confusing terms. So dental surgeons should also accept plainly speaking and meaning language in the treatment of this disease, so that its pathology may be clear and its therapeusis may be better and easier reached.

In my opinion, the inception of this disease is very often in uncleanliness. The catarrh has there its origin. We do not often see it, or at least do not recognize it, until it comes to us in its advanced stages, but could we see its beginnings, I have no doubt that the uncleaned gingival margins of the gums, covered with decomposing mucous and swarming with microbes, is the commencement of the difficulty. Dr. W. D. Miller in the *Independent Practitioner*, page 283, says upon this subject, quoting Von Kaczorowsky: "After several years experience and a long series of observations, he came to the conclusion that many cases of loss of appetite, nausea, dyspepsia, and consequent general ill health, were to be attributed to an unclean condition of the oral cavity, independently of the condition of the teeth themselves. Acting upon this conclusion, he was able to treat such cases with marked success by simply insisting upon repeated cleansing and sterilization of the mouth and throat."

He further says: "When we take into consideration that affections of the mucous membrane of the mouth may by continuity and contiguity spread over the whole mouth and throat, and thence upward and downward over the respiratory and digestive tracts, through the eustachian tube to the internal ear and brain, from the nose through the lachrymal canals to the eye, through the sieve of the ethmoid to the membranes of the brain: furthermore, when we remember that the irritation from the terminal branches of the quintus in the gums may produce irradiations in other branches of this nerve or irritations in near and distant organs of the body—when we keep all these things in mind and remember at the same time that many people, as Von Kaczorowsky rightly says, carry about constantly an amount of filth in the mouth which they would not tolerate for a moment upon the skin, then we can readily account for many troubles which otherwise appear so inexplicable and stubbornly resist every treatment by the usual internal remedies. Individuals who go about with neglect ed mouths, whose gums are swollen and suppurating, whose breath testifies to the intense fermentation going on in their unclean mouths, receive one prescription after another, or are sent from one bathing place to another, to no purpose whatever, when a thorough cleansing and disinfection of the mouth, accompanied with the necessary dental work, would restore them to health in a very short time."

So it is no doubt true that pyorrhœa or oral catarrh, instead of first commencing in the nose or throat, first is in the mouth from lack of care, and then communicates to the other tracts, as he says, "*per continuum et contiguum.*" The cases, however, when brought to us, are so advanced and the catarrhal condition so wide-spread that the start is not easily recognized.

Do we find further insight into the etiology of this disease in the study of bacteria, of its secretions? Not as yet. It is found a very difficult matter on account of the large number of different kinds to be found in the mouth and the difficulty of their cultivation.

In the recently published work, "The Annual of the Universal Medical Sciences," already referred to, the editor of the article on Pyorrhœa Alveolaris, Dr. Sudduth, M. D., F. R. M. S., writes as follows: "This is a term" (speaking of pyorrhœa) "applied to the secondary stages of a disease that has its inception in a catarrhal stomatitis. Being confined, as a rule to the margins of the gums surrounding the teeth, it might be called a 'gingivitis' were it not for the general catarrhal tendency shown by the entire mucous membrane of the mouth and nasal passages. The intimate relation between a general catarrhal idiosyncracy and pyorrhœa alveolaris is more than a mere coincidence. Its common occurrence in persons who have irregular teeth has also often been noted by the writer. He considers that this fact has, besides the matter of uncleanliness, a direct bearing upon its pathogeny. It is well known that the irregularities of the teeth present often an indication of a degenerative taint, and that persons in whom irregularities occur are very prone to catarrhal affections of the respiratory organs, including the nasal passages. Their skin is also usually found to be very susceptible to inflammatory affections. Another is the offensive odor of the saliva in individuals who show this peculiar tendency to catarrhal affections, even in persons who take the most scrupulous care of the teeth. In the majority of cases pyorrhœa is a form of stomatitis in which the local and constitutional factors in the production of the disease are largely dependent upon a hereditary catarrhal dyscrasia for their ability to engraft themselves upon the tissues."

Dr. Sudduth, in summing up, says: "The most common cause of catarrhal gingivitis is found in local irritation combined with some hereditary disposition to catarrhal affections. The next greatest etiological factor is symptomatic, the local manifestation of a constitutional vice. The most common manifestation is that of syphilis and its antidotes, mercury and iodide of potassium, both of which sometimes find expression in a localized inflammation, which may be the starting point for pyorrhœa alveolaris. As a complication of the disease in its secondary stages there can be no doubt of the action of micro-organisms. We do not feel justified, however, in conceding to them, as some do, a position of specificity." One word more as to pyorrhœa alveolaris being of constitutional origin and I will leave the etiology of the subject. My experience with the disease has shown me its presence in the mouths of patients who, while generally suffering from catarrh, have as a rule, been robust and healthy persons. I have never known a well developed symptom pointing to its origin in anæmia, tuberculosis, or concomitant with Bright's disease, urinary deposits, loss of health from high living, malarial fever, eruptive fevers, or syphilis. I do not deny—indeed, I admit—that a constitutional dyscrasia will retard or prevent a cure, but without local irritation these constitutional symptoms, in my opinion, can never initiate a case of pyorrhœa. Let me illustrate: A patient contracts syphilis while teeth and gums are in good condition, and who goes through medication with continued salivations, stands not the slightest danger of pyorrhœa if he persistently keeps his teeth clean, sleeps with his mouth shut—as a Christian should—and keeps undue irritants away from his gums; while on the other hand, no man or woman, healthy or unhealthy, is free from danger of pyorrhœa if they persistently neglect cleanliness, or breathe through the mouth and neglect catarrh which may be present in the nose, pharynx or some part of the alimentary or respiratory passages.

In closing this part of my paper on the etiology of pyorrhœa, I cannot refrain from my sense of satisfaction in the able manner in which Dr. Sudduth has corroborated my observations on the identity of pyorrhœa alveolaris and catarrh. I am proud to have found so distinguished an ally. I am also not forgetful of those here to-day who had kind words for my first effort upon this subject, and, so far as I know, are still of the same opinion. To my many critics, who have endeavored to refute my arguments in the papers first published, I have nothing to say, for not a single statement has been forwarded by them, which I deem worthy of debate. They have merely insisted that my catarrhal theory was wrong, but entirely failed to give scientific reason for their opinions.

I will now pass to the consideration of the treatment. My own treatment is simple and easily described. First, removing deposits, which, if they have not caused the disease, are prime factors in its later stages. In many cases much of the territory of the root which is affected is apparently free from calculus of any description, but careful examination will show minute cactus-like points, and the instrument must carefully pass over every part of the root affected which is or has been bathed in the secretions from the disease.

The key to success in all these cases lies, and must ever lie, in the thoroughness of the surgical procedure of removing all deposits upon the root. The instruments must be delicate, well adapted, and well tempered, and for the most part to be used with the push movement. I have always used Cushing's instruments, but the same work can be accomplished with other equally well adapted instruments. The territory must first be explored with a probe to prove its extent and deposit patches. With the expertness gained from experience I find there need be no failure to reach all surfaces liable to be affected and to remove the deposits. The most trying places to reach are to be found upon the inner surfaces of the roots of the superior molars. Yet, with patience and a knowledge of the particular anatomy of each individual tooth, which knowledge can be shown by probing, even these places can all be reached. If any part is neglected, a cure cannot be looked for. Those of us who have treated many cases cannot have failed to observe that in the healing process, if a slight point has been neglected, the bluish-red gum and the oozing pus will guide directly to it. My plan is, so far as possible, to operate upon one tooth at a time—not leaving it for another until

I am entirely satisfied that it is clean. It is no matter if the gums do bleed freely, you must in any event be guided by touch and the anatomical conformation of the root, and can work as well under profuse bleeding. I deprecate the partial cleaning of a number of teeth, then going to another part of the mouth and returning to those first operated upon. In that way territory is uselessly overhauled again and again, leaving uncertainty as to the operation at the last. Complete each tooth as you go; so that you will not have to return again at that operation, or at any subsequent operation.

Having the surgical treatment completed, the first thing next is to wash the *debris* of deposits and pus clean from the pockets and from the mouth with peroxide af hydrogen in an abscess syringe. This accomplished, the mouth is thoroughly rinsed again and again with sol. bichloride of mercury, and the deep pockets thoroughly syringed with the same.

The concluding treatment and the continued treatment is thorough cleaning and washing with "Listerine," according to the well-known directions of all the catarrhal surfaces. Listerine not being a germicide, I combine with the Listerine the solution of bichloride of mercury when I deem a germicide requisite. In the use of the douche for nasal catarrh, in addition to the Listerine, or, rather, preceding the Listerine, I use the following admirable preparation.

| | |
|---|---|
| Acid. carbolici . . . | gr j. |
| Sodæ biborat. . . . | |
| Sodæ bicarb. . . . | aa grs. ij. |
| Glycerine. . . . . | ʒ j. |
| Aquæ, ad. . . . . | ℥ j. |

It is known as "Dobell's solution," and is especially adapted to clearing the diseased surfaces of all viscid mucus and saliva preparatory to other medication.

When any of the teeth are so loose as to be moved considerably by the tongue or by mastication, they must be secured firmly in one position, or a cure is retarded and often entirely prevented. This is a very important step—more so than the medication, or any other step except surgical—and I implore you not to do this with ligatures, but strike up metal dies and swage to the inner surfaces of the teeth a narrow strip of gold plate. then make the bands for each of the teeth and solder to the plate. If the teeth are elongated, grind them off, or swage or solder a lug over the cutting, or grinding edge of the tooth to exert pressure, forcing the tocth into its socket. Do not let this plate reach the gum, but it should rest about half-way between the gum and cutting edge, thus affording easy cleansing of the teeth with brush, which would be prevented did the plate rest upon the gum or quite near it. This plate may if necessary, be frequently removed to afford a more ready cleaning. Thus the teeth are kept immovable in proper position, and the new tissue of repair is allowed to organize in the diseased pockets, which is prevented and an irritation kept up if the loose teeth are not thus secured.

I am very much opposed to continued and frequent medication of deep pockets. The plasma thrown out to organize into repair tissue must not be disturbed until sufficient time has elapsed to afford time for healing. If, however, after about three weeks, an irritation still seems to be present, further surgical interference must be given.—*Western Dental Journal.*

